# Optimising craniospinal irradiation for medulloblastoma: a dosimetric comparison of two VMAT planning methods

**DOI:** 10.3332/ecancer.2024.1700

**Published:** 2024-05-08

**Authors:** Niketa Thakur, Nancy Bansal, Meena Sudan, Abhishek Sharma

**Affiliations:** 1 Department of Radiation Oncology, All India Institute of Medical Sciences (AIIMS), Bilaspur 174037, India; 2 Department of Radiation Oncology, Sri Guru Ram Das Institute of Medical Sciences and Research, Amritsar 143501, India; 3 Department of Anesthesia and Critical Care, Sri Guru Ram Das Institute of Medical Sciences and Research, Amritsar, India

**Keywords:** craniospinal irradiation, volumetric modulated arc therapy (VMAT), avoidance sector, partial arc

## Abstract

**Background:**

Craniospinal irradiation (CSI) poses a challenging planning process because of the complex target volume. Traditional 3D conformal CSI does not spare any critical organs, resulting in toxicity in patients. Here the dosimetric advantages of volumetric-modulated arc therapy (VMAT) using partial arc and avoidance sectors are compared with each other in planning in adult patients undergoing CSI to develop a clinically feasible technique that is both effective and efficient.

**Patient and methods:**

Eight adult patients treated with CSI were retrospectively identified. In total 16 plans were made. We generated two plans for each patient: 1. VMAT plan using partial arc, namely VMAT_pa. 2. VMAT plan using avoidance sectors, namely VMAT_as. The dose prescribed was 36 Gy in 20 fractions. The dose-volume histogram for planning target volume (PTV) and organs at risk (OAR) (lens, eye, heart, thyroid, lungs, liver, gonads and kidneys) were analysed and compared. Dose parameters of mean dose, V_95%_, and V_107%_ for the PTV were evaluated.

**Results:**

The median length of PTV is 65.58 cm (45.8–79.5). The volume of PTV receiving 95% of the dose (V95%) in both the plans are 97.51% (VMAT_as) and 97.99% (VMAT_pa) (*p* = 0.121) while V107% are 0.733 and 0.742 for VMAT_as and VMAT_pa, respectively (*p* = 0.969). The doses of OARs such as lens, eye, liver and gonads were comparable. The mean heart dose was 10.4 and 9.0 Gy in VMAT_as and VMAT_pa plans, respectively (*p* = 0.005). Significant lower doses to the thyroid, kidneys and lungs were seen in VMAT plans using avoidance sectors.

**Conclusion:**

This study provides a practically useful VMAT planning method for the treatment of CSI and illustrates the ability of VMAT using avoidance sectors to generate highly conformal and homogeneous treatment plans for CSI, while limiting the dose to the relevant OARs.

## Introduction

The goal of craniospinal irradiation (CSI) is to treat the entire craniospinal axis, which includes the brain and the full length of the spinal cord. The primary challenge in CSI is to adequately cover the target while minimising the dose to the surrounding organs at risk (OAR), such as the heart, lungs, thyroid and so on. There has been a growing interest in the use of volumetric modulated arc therapy (VMAT) for CSI due to its dosimetric advantages which include shorter treatment times compared to conventional intensity-modulated radiotherapy (IMRT), increased dose conformity, and the potential to reduce side effects. However, it requires careful planning and advanced technology to implement effectively.

Full-arc VMAT can deliver radiation quickly and efficiently; however, it may pose challenges in protecting OARs from radiation exposure [1]. Partial-arc VMAT can better protect OARs by directing the radiation beam more precisely to the target area [2]. However, the treatment delivery time might be longer than full-arc VMAT. Avoidance sectors can further enhance the sparing of OARs. This approach designates certain areas to be avoided by the radiation beam, thereby protecting critical structures [3]. Several studies have demonstrated that both partial-arc and avoidance sector techniques in VMAT for CSI can lead to better sparing of OARs such as the heart, thyroid, and lungs compared to full-arc VMAT [4]. They still maintain excellent target coverage and dose conformity.

Combining partial arcs and avoidance sectors in VMAT planning for adult CSI might offer an effective and efficient treatment method, balancing excellent tumour coverage with minimised exposure to OARs. Some studies have highlighted the use of VMAT for CSI, specifically focusing on the use of partial arcs and avoidance sectors. Ghandour *et al* [5] described the benefits of VMAT with avoidance sectors in protecting OARs during the treatment of prostate cancer. Similar strategies could be applied to CSI to minimize dose to adjacent OARs. Many studies have demonstrated the utilisation of avoidance sectors in VMAT planning to minimize radiation exposure to the OARs, a technique that could be adapted for the avoidance of critical structures in CSI as well [6–7].

Keeping this in the background, we aimed to study the dosimetric advantages of VMAT using partial arc and avoidance sectors in planning in adult patients undergoing CSI to develop a clinically feasible technique that is both effective and efficient in terms of target coverage and normal tissue sparing.

## Materials and methods

### Patient immobilisation and simulation

Eight adult patients treated with CSI were retrospectively identified. All the patients had undergone tumour resection before the start of radiotherapy. Each patient underwent a simulation computed tomography (CT) scan of 3 mm slice thickness in head first supine position with both arms resting by the side of the body. Thermoplastic masks tailored to the head neck region and pelvis were used to ensure proper immobilisation and high reproducibility.

### Target and OAR delineation

CT images were transferred to the Monaco treatment planning system (version 5.11.03) for target volume and OAR contouring and subsequent treatment planning. The OAR contoured on the image data sets were eyes, lens, lungs, heart, thyroid, liver, kidneys and gonads. Furthermore, the cranial portion of the clinical target volume (CTV), encompassing the entire brain, cranial nerves and meninges, was delineated. The caudal portion of the CTV encompasses the entire subarachnoid area from the foramen magnum to the lower aspect of the thecal sac, as well as a lateral expansion that includes the nerve roots, in accordance with the SIOPE guideline [8].

### Treatment planning

The treatment planning system was done using Monaco 5.11.03 planning software and calculation was done with Monte Carlo Algorithm. A total of 16 plans were made. We generated two plans for each patient: 1. VMAT plan using partial arc, namely VMAT_pa. 2. VMAT plan using avoidance sectors, namely VMAT_as. Most of the plans had three isocentres. However, if the combined craniocaudal length of the brain and spine planning target volume (PTV) was less, two isocenters were employed. Dose optimisation constraints for all the OARs in the inverse planning process were set for each patient individually. The optimisation criteria included grid spacing of 0.30 cm and statistical uncertainty of 1%. The optimisation for both PTVs was done simultaneously with the overlapping area between the fields of the two isocenters ranging between 2 and 4 cm.

### VMAT planning with partial arcs

**Figure 1.**
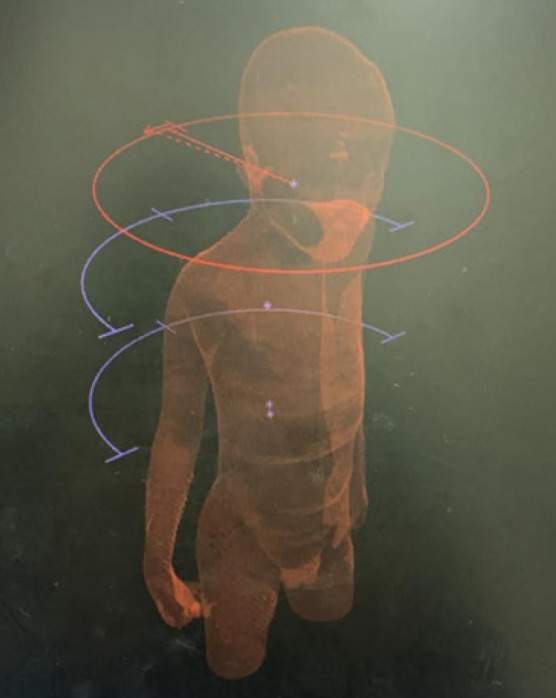
Arc arrangements for the VMAT_pa technique. For the upper part of the PTV (brain and upper spine), two coplanar arcs in clockwise and counterclockwise direction are used. For the lower part of the PTV two 90 degrees arcs in clockwise direction and two 90 degrees arcs in counter clockwise direction are used.

To cover the upper part of the PTV (brain and upper spine), two coplanar arcs with opposite directions of rotation (clockwise: CW, counterclockwise: CCW) were used. For the lower part of the PTV, two 90-degree arcs in a clockwise direction and two 90-degree arcs in counter clockwise direction were used as shown in Figure 1. The plans did not involve any beam entry through the arms.

### VMAT planning with avoidance sectors

**Figure 2.**
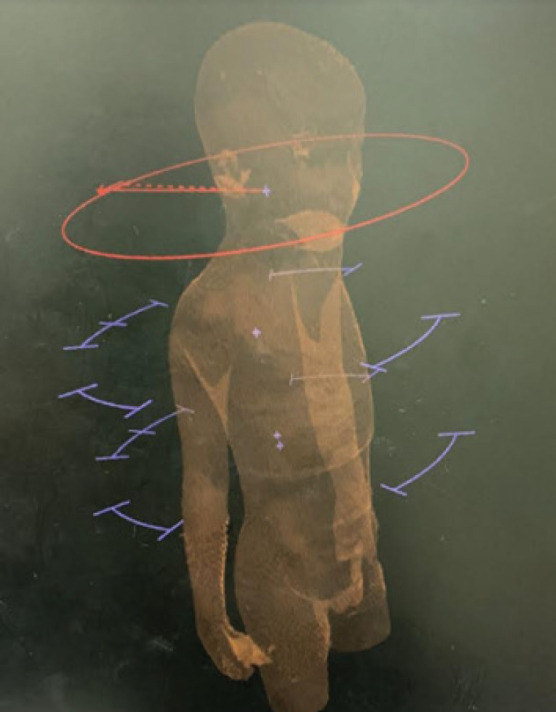
Arc arrangements for the VMAT_as technique. Two coplanar arcs are employed in both clockwise and counterclockwise directions for treating the upper part of the PTV, which encompasses the brain and upper spine. Meanwhile, 40-degree avoidance sectors are utilised for treating the lower part of the PTV, i.e., the lower spine. In total, six quarter arcs are used.

For the upper part of the PTV (brain and upper spine), two coplanar arcs with opposite directions of rotation (clockwise: CW, counterclockwise: CCW) were used. For the lower part of the PTV (lower spine), 40 40-degree avoidance sectors were used as shown in Figure 2. A total of six quarter arcs were used. None of the plans included a beam entry via the arms.

### Plan evaluation

Dose parameters of mean dose, V_95%_, and V_107%_ for the PTV were evaluated. The dose-volume histogram (DVH) for PTV and OAR (lens, eye, heart, thyroid, lungs, liver, gonads and kidneys) were analysed and compared. For the lungs, different volume characteristics like the V5, V10 and V20 (volume of the lungs receiving doses of 5, 10 and 20 Gy) were evaluated.

The conformity index (CI) was used to quantify planned dose conformity. CI was calculated as follows [9]:

CI = TV_PIV_^2^/(TV*PIV).

Here, TV_PIV_ is the volume of target volume covered by prescription isodose, TV is target volume and PIV is prescription isodose volume.

The CI ranges from 0 to 1. A CI value close to 1 indicates enhanced PTV conformity.

According to ICRU report No.83, the homogeneity index (HI) was calculated as follows with an HI of 0 indicating excellent plan:

HI = (D2%−D98%)/D50%.

D98% is the dose received by 98% volume of the PTV, D50% is the dose received by 50% volume of the PTV and D2% is the dose received by 2% volume of the PTV.

### EBRT dose prescription and delivery

External beam radiotherapy dose to the PTV was given as 36 Gy/20 fractions, 1.8 Gy/fraction and five fractions/week. Image guidance with daily cone-beam CT was performed to verify the treatment setup.

### Statistical analysis

The data were entered into a Microsoft Excel spreadsheet and analysed using IBM SPSS Statistics for Windows, version 24 (IBM Corp., Armonk, NY, USA). A student’s paired *t*-test was performed to interpret the results, and a *p*-value of less than 0.05 was considered statistically significant.

## Results

The median length of PTV is 65.58 cm (45.8–79.5). The volume receiving 95% of the dose (V95%) of PTV in both the plans are 97.51% (VMAT_as) and 97.99% (VMAT_pa) (P=0.121) while V107% are 0.733 and 0.742 for VMAT_as and VMAT_pa, respectively (*p* = 0.969). The dosimetric differences for PTV parameters are mentioned in Table 1.

The mean values of CI are 0.860 ± 0.063 and 0.850 ± 0.045 in VMAT_as and VMAT_pa plan (*p* = 0.675), respectively, while mean HI values are 0.095 ± 0.003 and 0.099 ± 0.004 respectively in VMAT_as and VMAT_pa plan (*p* = 0.845).

The dosimetric differences for several OARs are highlighted in Table 2.

The maximum lens doses were comparable in both the treatment groups (Right lens (8.34 and 8.84 Gy in VMAT_as and VMAT_pa, respectively) *p* = 0.548 and Left lens (8.80 and 8.56 Gy in VMAT_as and VMAT_pa, respectively) *p* =0.761). The mean heart dose was 10.4 and 9.0 Gy in VMAT_as and VMAT_pa plans, respectively (*p* = 0.005). The mean doses to thyroid were 16.10 and 16.37 in VMAT_as and VMAT_pa, respectively (*p* = 0.030), while for liver were 7.31 and 6.68 (*p* = 0.236). The mean kidney doses were significantly less in VMAT_as plan (Right kidney (11.36 and 12.72 Gy in VMAT_as and VMAT_pa, respectively) *p* = 0.020 and left kidney (11.99 and 12.56 Gy in VMAT_as and VMAT_pa, respectively) *p* = 0.045).

**Figure 3.**
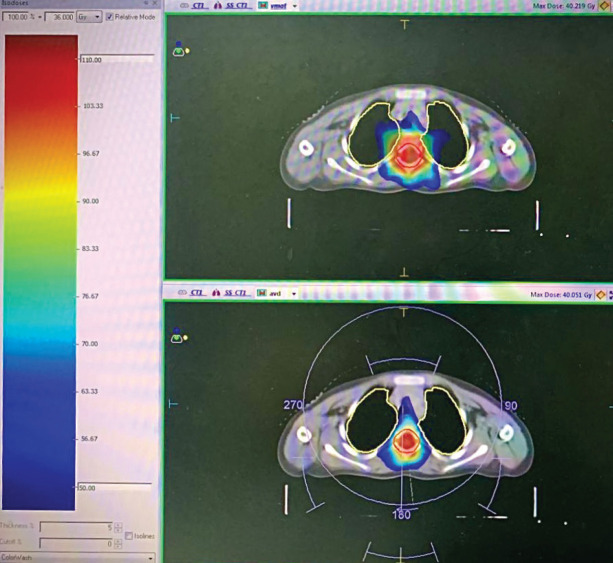
Colour wash showing axial dose distribution. There is no dose to lungs with 50% isodose using VMAT plan with avoidance sectors (VMAT_as).

**Figure 4.**
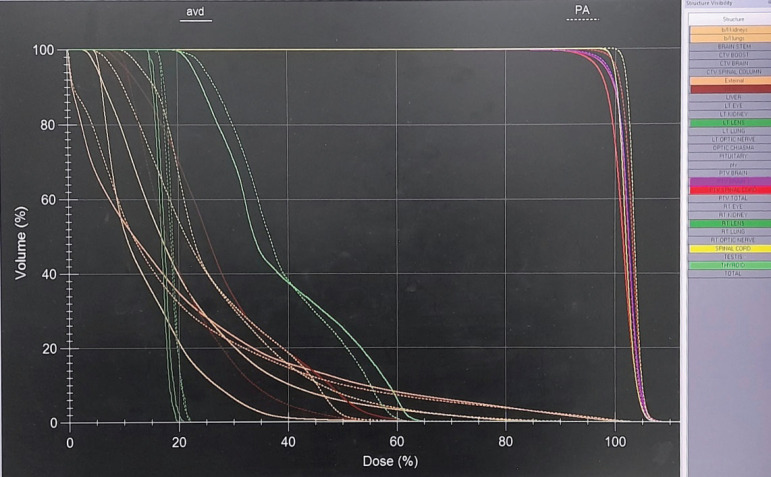
DVHs for VMAT_as (using avoidance sectors) and VMAT_pa (using partial arcs). Reduced dose to organs such as lungs, kidneys and thyroid with VMAT_as plan can be seen.

A significant difference in doses was seen in the lungs as Dmean, V5, V10 and V20 to lungs were significantly less with VMAT_as plan (Table 2). The lung Dmean doses were 8.8 Gy in VMAT_as and 10.6 Gy in VMAT_pa plans (*p* = 0.049). In VMAT_as plan, lung V5 and V10 were 69.7% and 27.4%, respectively, while they were 84.2% and 42.8% in VMAT_pa plan (*p* = <0.001 and 0.004 for V5 and V10, respectively). The lungs receiving 20 Gy (V20) were 2.4% and 9.0% in VMAT_as and VMAT_pa plans, respectively (*p* = 0.013). Figure 3 shows less axial dose distribution to lungs using the VMAT plan with avoidance sectors. The dosimetric differences for the individual organs at risk are presented in a Dose volume histogram for one patient in Figure 4.

Monitor Units (Mus) were 1,340 and 1,377 in VMAT_as and VMAT_pa plans, respectively (*p* = 0.455).

## Discussion

Due to its impact on patients’ long-term survival, CSI planning is widely regarded as one of the most intricate procedures in radiation planning [10–12]. This study’s objective was to compare the dosimetry between two different VMAT planning methods using partial arcs and avoidance sectors in patients of medulloblastoma undergoing CSI. The dosimetric results illustrated that a homogeneous and conformal dose to the brain and spinal canal could be achieved, while limiting the dose to the relevant OARs, with VMAT planning using avoidance sectors. There are a number of studies that have explored the use of VMAT planning in CSI.

An essential goal in CSI is to attain optimal target coverage while preserving uniformity. VMAT allows for the attainment of precise and uniform dose distributions which can achieve highly conformal dose distributions with improved target volume coverage and sparing of normal tissues compared with conventional radiotherapy techniques [13–15]. VMAT also offers additional advantages, such as reduced treatment delivery time compared with static field IMRT. The use of parameters is contingent upon the utilisation of distinct weightings and priorities in the planning process. In our study, both planning methods demonstrated comparable target coverage along with comparable CI and HI.

Minimising treatment-related toxicities is crucial in paediatric patients, and it is of utmost importance to spare OAR. Lee *et al* [16] conducted a comparison between a Smart arc-based VMAT approach and conventional irradiation for CSI (23.4 Gy/13 portions). The researchers found that the median CI was 1.22 (range: 1.09–1.45) and 1.04 (range: 1.03–1.07). Additionally, there was a notable decrease in the average and maximum doses to the heart, thyroid, oesophagus, optic nerves and eyes when compared to conventional techniques.

Studies have examined conventional IMRT and tomotherapy in the context of CSI [3, 17–19]. In their study, Seppälä *et al* [17] devised a technique that utilises dynamic split-field IMRT with a single plan to enhance dose homogeneity in the target volume of CSI. The outcomes demonstrated that the split-field IMRT provided more consistent dose distribution in the target volume than the conventional 3DCRT.

In our study, the mean CI and HI are comparable in both treatment groups. The comparison between the two planning methods revealed notable differences in terms of OAR sparing. VMAT plans with the avoidance sector method exhibited superior sparing of organs with comparable doses to other organs attributed to its ability to actively avoid these regions during irradiation. VMAT plans with avoidance sectors showed a significant reduction of doses to OARs such as thyroid, lungs and kidneys while doses to the lens, eyes, liver and gonads are comparable in both treatment groups. Lung Dmean, V5, V10 and V20 to lungs were significantly less with VMAT plans using avoidance sectors. This may contribute to a reduced risk of late complications, emphasising its potential superiority in terms of long-term treatment-related side effects.

Secondary malignancies in CSI are becoming an increasing concern, particularly for paediatric patients. VMAT may allow for reduced doses of radiation administered to the thyroid, heart and ovaries, according to studies. Survivors of childhood cancer who underwent radiotherapy are more susceptible to the development of hypothyroidism. Moreover, as the cumulative irradiation dose to the organ increases, so does the associated risk [20]. The VMAT method is specifically engineered to optimise the utilisation of MUs, thereby reducing the gravity of this issue.

In contrast to conventional IMRT, VMAT may therefore contribute to a reduction in the overall risk of secondary malignancies among patients with a lengthy life expectancy. Pollul *et al* [21] reported the mean doses for the VMAT plan using avoidance sectors as 6.6, 8.7 and 1.9 Gy for the heart, thyroid, and gonads, respectively, in comparison to the 3D-CRT treatment procedure. Mean thyroid doses were considerably lower in our study for VMAT plans utilising avoidance sectors, whereas gonad doses were comparable.

The clinical implications of these dosimetric findings should be carefully considered when determining the most appropriate VMAT planning method for CSI in medulloblastoma patients. While both techniques meet the standard requirements for target coverage, the choice between partial arcs and avoidance sectors should be tailored to individual patient characteristics, treatment goals, and the importance of minimising long-term toxicities.

It is essential to acknowledge the limitations of this study, such as the small sample size and the inherent variability in patient anatomy. Future investigations with larger cohorts and longer follow-up periods are warranted to validate the dosimetric findings and assess the clinical outcomes associated with each planning method. Additionally, the impact of intrafraction motion and setup uncertainties should be considered in future studies to enhance the robustness of the results.

## Conclusion

This study provides a practically useful VMAT planning method for the treatment of CSI and illustrates the ability of VMAT using avoidance sectors to generate highly conformal and homogeneous treatment plans for CSI, while limiting the dose to the relevant OARs. The study highlights the potential of VMAT using avoidance sectors to achieve superior normal tissue sparing without compromising target volume coverage. Therefore, it can be considered as a promising option for planning in patients of CSI.

## Conflicts of interest

There are no conflicts of interest.

## Funding

No financial support was received from any organisation for the submitted work.

## Ethical approval

The research is hereby approved by the Institutional Ethics Committee.

Sri Guru Ram Das University of Health Sciences Institutional Ethics Committee issued approval.

## Author contributions

**Table:** 

	Author 1	Author 2	Author 3	Author 4
Concepts	✓			
Design	✓	✓		
Definition of intellectual event	✓	✓	✓	
Literature search	✓	✓		
Clinical studies	✓	✓		
Experimental studies	✓	✓	✓	
Data acquisition	✓	✓	✓	
Data analysis	✓	✓	✓	
Statistical analysis	✓	✓		✓
Manuscript preparation	✓			
Manuscript editing	✓	✓		✓
Manuscript review	✓	✓		✓
Gaurantor	✓			

## Figures and Tables

**Table 1.** DVH comparisons for PTV.

PTV parameters	VMAT_avd	VMAT_pa	*p*-value
CI	0.86 ± 0.063	0.85 ± 0.045	0.675
HI	0.095 ± 0.003	0.099 ± 0.004	0.845
V95	99.51	99.99	0.121
V107	0.73	0.74	0.969
MU	1340	1377	0.455

DVH, Dose volume histogram; PTV, Planning target volume; D mean, Mean dose; CI, Conformity index; HI, Homogeneity index; V95, Volume of PTV receiving 95% of the prescription dose; V107, Volume of PTV receiving 107% of the prescription dose

**Table 2.** DVH comparison for OAR.

Organs		VMAT_as	VMAT_pa	*p*-value
Eye (Dmax)	Right	33.13	32.15	0.144
	Left	33.50	33.16	0.588
Lens (Dmax)	Right	8.34	8.84	0.548
	Left	8.80	8.56	0.761
Thyroid (Dmean)		16.10	16.37	0.030
Heart (Dmean)		10.42	9.03	0.005
Lungs (Dmean)		8.84	10.62	0.049
V 5 Gy		69.72	84.22	<0.001
V 10 Gy		27.48	42.89	0.004
V 20 Gy		2.45	9.00	0.013
Liver (Dmean)		7.31	6.68	0.236
Kidney (Dmean)	Right	11.36	12.72	0.020
	Left	11.99	12.56	0.045
Gonads (Dmean)		0.20	0.21	0.621

DVH, Dose volume histogram; OAR, Organ at risk
